# Discovery of Bacterial Fimbria–Glycan Interactions Using Whole-Cell Recombinant Escherichia coli Expression

**DOI:** 10.1128/mBio.03664-20

**Published:** 2021-02-23

**Authors:** Christopher J. Day, Alvin W. Lo, Lauren E. Hartley-Tassell, M. Pilar Argente, Jessica Poole, Nathan P. King, Joe Tiralongo, Michael P. Jennings, Mark A. Schembri

**Affiliations:** a Institute for Glycomics, Griffith University, Gold Coast, Queensland, Australia; b School of Chemistry and Molecular Biosciences, The University of Queensland, Brisbane, Australia; c Australian Infectious Diseases Research Centre, The University of Queensland, Brisbane, Australia; The Ohio State University School of Medicine

**Keywords:** *Escherichia coli*, FimH, glycomics, fimbriae, glycoproteins

## Abstract

Chaperone-usher (CU) fimbriae are the most abundant Gram-negative bacterial fimbriae, with 38 distinct CU fimbria types described in Escherichia coli alone. Some E. coli CU fimbriae have been well characterized and bind to specific glycan targets to confer tissue tropism. For example, type 1 fimbriae bind to α-d-mannosylated glycoproteins such as uroplakins in the bladder via their tip-located FimH adhesin, leading to colonization and invasion of the bladder epithelium. Despite this, the receptor-binding affinity of many other E. coli CU fimbria types remains poorly characterized. Here, we used a recombinant E. coli strain expressing different CU fimbriae, in conjunction with glycan array analysis comprising >300 glycans, to dissect CU fimbria receptor specificity. We initially validated the approach by demonstrating the purified FimH lectin-binding domain and recombinant E. coli expressing type 1 fimbriae bound to a similar set of glycans. This technique was then used to map the glycan binding affinity of six additional CU fimbriae, namely, P, F1C, Yqi, Mat/Ecp, K88, and K99 fimbriae. The binding affinity was determined using whole-bacterial-cell surface plasmon resonance. This work describes new information in fimbrial specificity and a rapid and scalable system to define novel adhesin-glycan interactions that underpin bacterial colonization and disease.

## INTRODUCTION

Fimbriae (also known as pili) are proteinaceous structures that extend from the surface of many bacteria. These organelles mediate diverse functions associated with the colonization of surfaces and virulence, including adherence and biofilm formation. Most bacterial fimbriae are comprised of a major repeating protein that makes up the bulk of the organelle as well as a tip-located adhesin. The adhesin typically recognizes specific receptor targets in a lock-and-key fashion, thereby determining tissue tropism.

Many different types of fimbriae have been described in Gram-positive and Gram-negative bacteria (1, 2). In Gram-negative bacteria, fimbriae assembled by the chaperone-usher (CU) system are the most abundant, with CU fimbriae of Escherichia coli being the best characterized (3). The prototypical CU fimbriae are type 1 and P fimbriae from uropathogenic E. coli (UPEC), and their regulation, biogenesis, structure, and function have been extensively studied (3–7). Type 1 fimbriae extend ∼1.0 μm from the cell surface and are composed of a major subunit (FimA) as well as a tip fibrillum comprising several minor components, including the FimH adhesin (8–10). FimH binds to α-d-mannosylated glycoproteins, such as uroplakins, that are abundant in the bladder (11), thereby facilitating UPEC colonization and invasion of the bladder epithelium (12, 13). P fimbriae adopt a similar overall structure, comprising a major structural protein (PapA), which makes up the bulk of the organelle that is connected to a tip fibrillum composed of major (PapE) and minor (PapF, PapK, and PapG) subunits (14). The PapG adhesin is located at the distal tip of the fibrillum and binds to Galα(1-4)Gal-containing glycolipids (15). Three classes of PapG adhesin have been described with respect to binding affinity. The class I adhesin binds to globotriaosyl ceramide (GbO_3_), the class II adhesin binds to globotetraosyl ceramide (GbO_4_), and the class III adhesin binds to the Forssman glycolipid with a terminal GalNAc (GbO_5_) (15, 16). The class II PapG allele is the most common type of adhesin found in UPEC strains that cause pyelonephritis and is essential for colonization of the upper urinary tract in a nonhuman primate infection model (17).

The analysis of whole-genome sequence data has revealed extraordinary diversity in CU fimbriae at the genetic level (18). Indeed, 38 distinct CU fimbrial types have been identified in E. coli alone based on the phylogeny of the conserved usher protein and genome locus position (19). This variation is also reflected in the receptor specificity of the tip-adhesin (or lectin) to specific glycans (20) and dictates targeted adherence properties associated with different E. coli pathotypes. For example, CU fimbriae, including P, F1C, and S, are frequently associated with UPEC and meningitis-associated E. coli (NMEC) (17, 21, 22), aggregative adherence fimbriae (AAF) are associated with enteroaggregative E. coli (EAEC) (23), long polar fimbriae (LPF) are associated with enteropathogenic E. coli (EPEC) and enterohemorrhagic E. coli (EHEC) (24), CS1-CFA/I are associated with human-enterotoxigenic E. coli (ETEC) (25), and K88 (F4) and K99 (F5) fimbriae are associated with porcine, bovine, and ovine ETEC (26, 27). Most E. coli strains also contain various combinations of CU fimbrial genes (19, 28), and these fimbriae can work in concert to dictate tropism to specific infection sites. Recent work has shown that the expression of Ucl fimbriae (also known as F17-like fimbriae) (29) contributes to UPEC colonization of the gut, enabling the formation of a reservoir for subsequent infection of the urinary tract due to the expression of type 1 fimbriae (30). Furthermore, understanding these interactions at the molecular level represents an exciting approach for the development of new anti-adhesion molecules as alternative treatments to disrupt colonization by multidrug-resistant pathogens (30–34).

Despite our knowledge of CU fimbria diversity and the contribution of selected fimbriae to pathogenesis, many CU fimbriae remain to be properly characterized. In addition, a comprehensive understanding of their receptor specificity is lacking. In this study, we developed a new approach to dissect CU fimbria binding using a glycan array. First, we showed that the glycan binding profile of the purified FimH adhesin lectin-binding domain (FimH^LD^) and a recombinant E. coli strain expressing type 1 fimbriae are similar, demonstrating that rapid screening of bacteria expressing a single type of CU fimbriae can be employed to dissect receptor specificity. Next, a series of plasmids containing genes encoding additional 12 CU fimbriae, including P (representing the three PapG allelic variants), F1C, Afa, F9, Yqi, Mat (also known as Ecp), type 3, K88 (AB and AC types), and K99, were transformed into the E. coli K-12 *fim*-negative strain MS428, and these recombinant strains were screened for binding to specific glycans. Overall, we were able to define a set of receptor-binding phenotypes for each CU fimbria, many of which matched their specific pathotype association.

## RESULTS AND DISCUSSION

### The purified FimH lectin-binding domain and recombinant cells expressing type 1 fimbriae interact with similar glycans based on glycan array screening.

**FIG 1:**
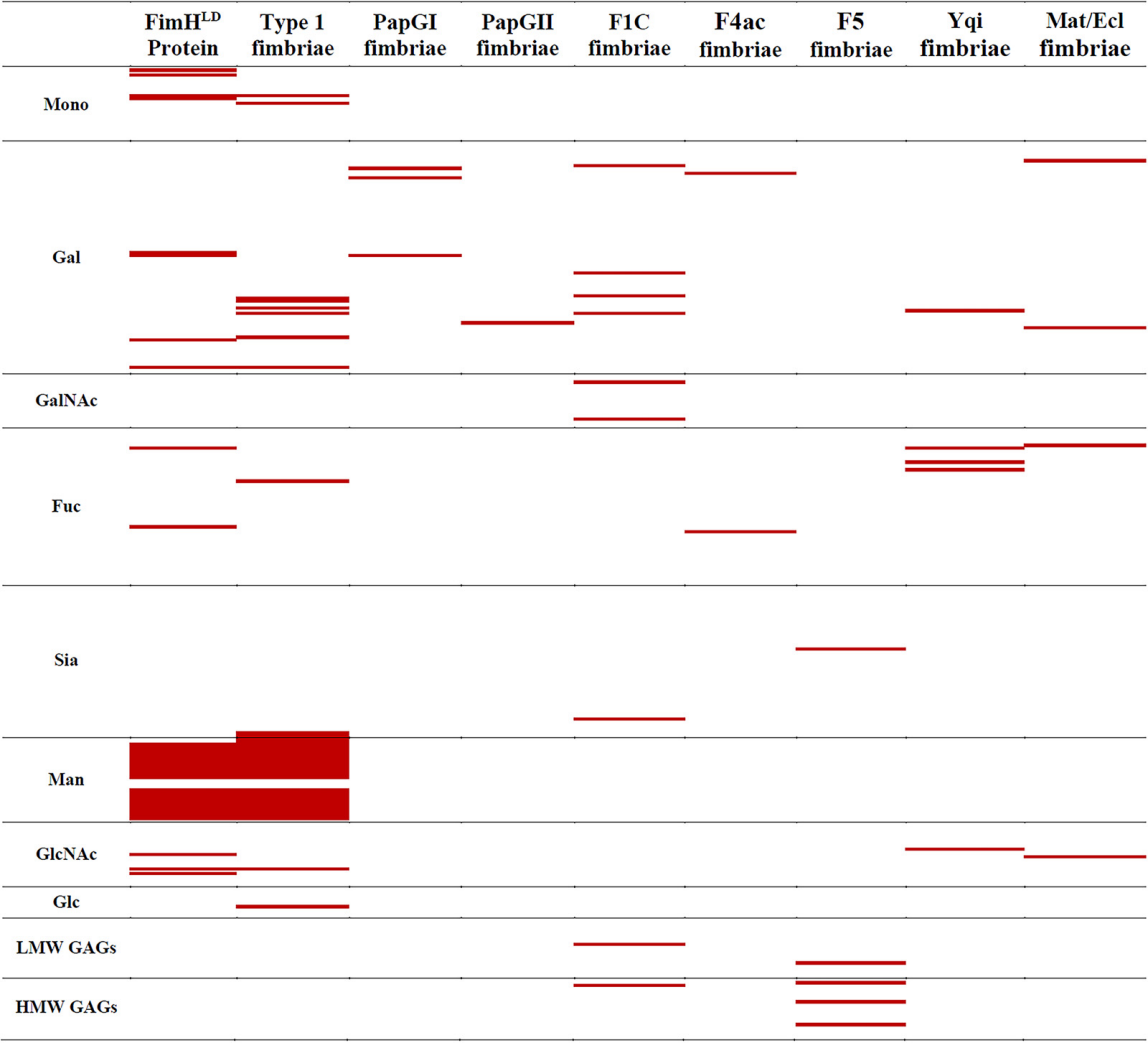
Glycan array analysis of a range of fimbrial proteins from E. coli. This figure provides a graphical representation of the pattern of binding of fimbrial proteins to the 375 glycans present on the Institute for Glycomics glycan microarray to identify similarities and differences in binding patterns between these proteins. Full data showing the identity of each glycan bound is shown in Data Set S1 and is discussed in detail in the text. Red indicates binding above background; white indicates no binding. Mono, monosaccharides; Gal, terminal galactose; GalNAc, terminal N-acetylgalactosamine; Fuc, fucose-containing glycans; Sia, sialylated glycans; Man, mannose-containing glycans; GlcNAc: terminal *N*-acetylglucosamine; Glc, repeating glucose; LMW GAGs, low-molecular-weight glycosaminoglycans; HMW GAGs, high-molecular-weight GAGs.

Methods to elucidate and define protein-glycan interactions often start with the use of recombinant purified proteins to screen glycan targets on arrays. However, such approaches are not amenable to high-throughput investigation due to the requirement for purified protein. Furthermore, recombinant purified protein domains may not correspond to natively expressed fimbrial protein on the bacterial cell surface. Therefore, we began by examining the glycan binding repertoire of type 1 fimbriae as a model, well-characterized system using two parallel approaches: (i) purified functional FimH^LD^ protein fused at the C terminus to a 6× histidine tag (35) and (ii) a recombinant E. coli K-12 *fim* deletion strain (MS428) (36) transformed with a plasmid containing the *fimAICDFGH* gene cluster (pPKL4) (37). In general, purified FimH^LD^ and the recombinant type 1 fimbria-expressing MS428(pPKL4) strain bound to a very similar group of related and overlapping glycans on the array (Fig. 1; see also Data Set S1 in the supplemental material). This encompassed a broad range of mannose-containing glycans, including α-mannose monosaccharide, α1-2-, 3-, 4-, and 6-linked mannobiose, and branched mannose structures, such as Man5 (Fig. 1 and Data Set S1). Several glycan structures not terminating in mannose were also bound by either FimH^LD^ or MS428(pPKL4), suggesting that there are minor conformational differences that could explain the small variance in binding between the purified FimH^LD^ protein and natively expressed FimH integrated at the tip of the type 1 fimbria structure. Indeed, FimH has been shown to adopt both low- and high-affinity binding conformations that could explain this phenomenon (38). No binding to the glycan array was observed by MS428 harboring the empty vector (Data Set S1), demonstrating that the glycan interactions were type 1 fimbria dependent. Overall, the binding to mannose terminating structures by FimH^LD^ and MS428(pPKL4) agrees strongly with the published binding profile of the FimH adhesin (39), supporting an approach employing recombinant whole cells to determine the glycan binding profile of a specific type of fimbriae.

10.1128/mBio.03664-20.1 Data Set S1 Glycan array analysis of a range of fimbrial proteins from E. coli. This dataset provides the information of the glycan binding of fimbrial proteins to the 375 glycans present on the Institute for Glycomics glycan microarray presented in Fig. 1. Red indicates binding above background; white indicates no binding. Mono, monosaccharides; Gal, terminal galactose; GalNAc, terminal N-acetylgalactosamine; Fuc, fucose-containing glycans; Sia, sialylated glycans; Man, mannose-containing glycans; GlcNAc, terminal N-acetylglucosamine; Glc, repeating glucose; LMW GAGs, low-molecular-weight glycosaminoglycans; HMW GAGs, high-molecular-weight GAGs. Download Data Set S1, PDF file, MB. Copyright © 2021 Day et al. 2021 Day et al. https://creativecommons.org/licenses/by/4.0/ This content is distributed under the terms of the Creative Commons Attribution 4.0 International license.

### SPR can be used to accurately measure whole-cell–glycan interactions.

**FIG 2:**
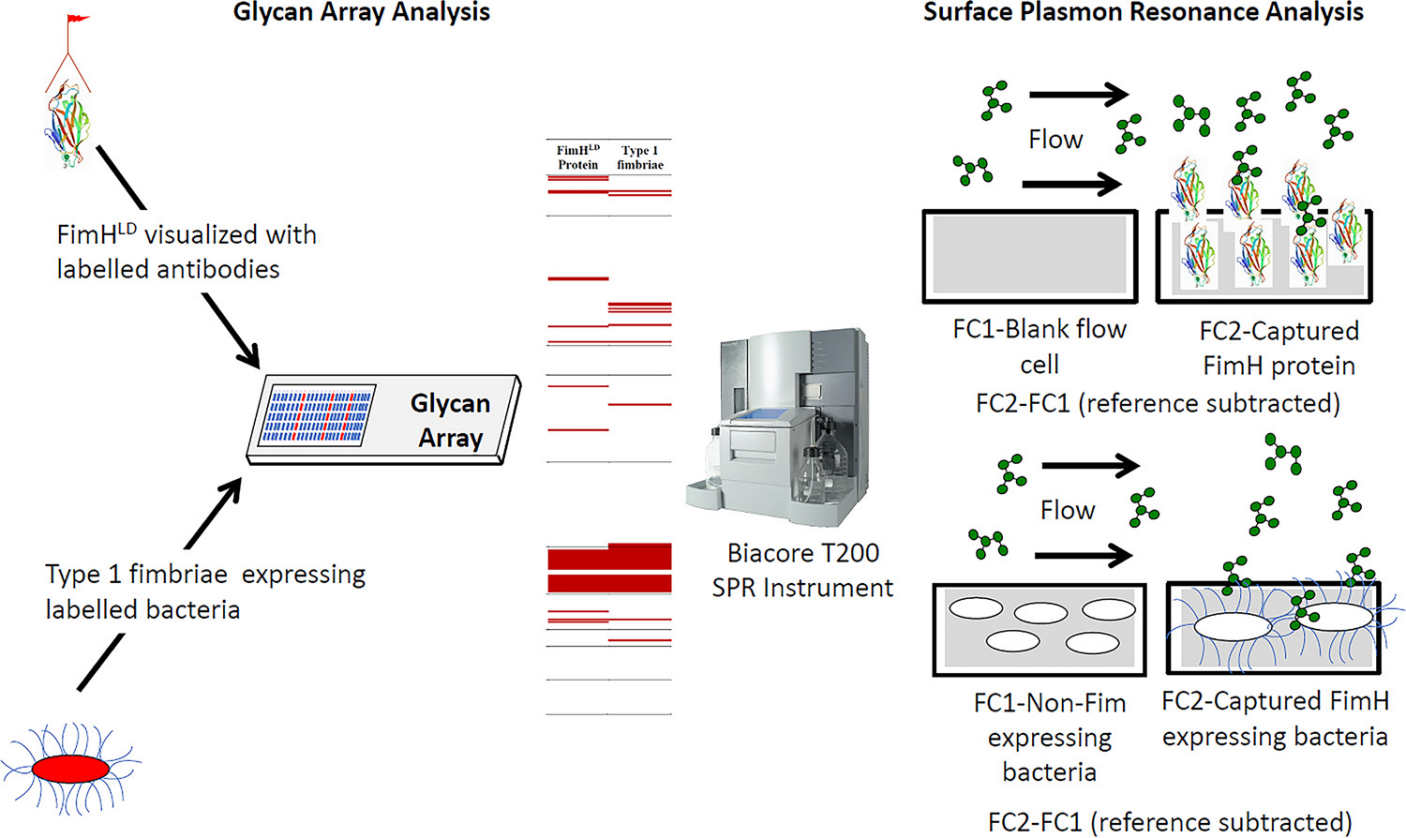
Workflow of the comparison between purified FimH^LD^ and type 1 fimbria-expressing E. coli. From left to right, glycan array analysis compares the purified protein detected with fluorescent antibodies (red) to fluorescent dye-labeled bacteria (red). This provides fluorescent signals on the array that can be detected and presented as a yes/no binding across 400 glycans. Surface plasmon resonance analysis takes the positive binding and allows for the determination of the affinity (*K_D_*) using either the purified protein (CM5 chip, high-density dextran layer) or the whole bacteria (C1 chip, no dextran layer, binding very close to the gold surface). Each analysis requires a blank flow cell (FC1) with an ethanolamine-blocked dextran layer used for protein and immobilized MS428 (*fim* negative) for the type 1 fimbria-expressing strain.

To extend our comparative analysis of FimH^LD^ and MS428(pPKL4) binding to glycans, we used surface plasmon resonance (SPR) to quantitate their binding affinity to both Man5 and Man5GlcNAc (Fig. 2). In these experiments, FimH^LD^ bound to both mannosylated glycans with high affinity (Table 1), and the dissociation constant (*K_D_*) values were consistent with previously published affinity data for FimH and these two glycan structures (110 to 127 nM for Man5 and 12 to 20 nM for Man5NAc) (39). Next, we adapted a previously described method that employed SPR to measure toxin-mammalian cell receptor binding (40) to quantitate the interaction between MS428(pPKL4) cells and both glycans (Table 1). These data were congruent with the affinity data obtained using FimH^LD^, further supporting the use of recombinant whole cells expressing a specific fimbria type to precisely quantitate individual glycan interactions.

**TABLE 1:** Surface plasmon analysis of fimbrial proteins

Compound	Disassociation constant^*a*^:			
	FimH (protein)	FimH (E. coli)	F1C (E. coli)	K88 (E. coli)
Man5	607 ± 142 nM	2.0 ± 0.842 μM	NCDI	NCDI
Man5NAc	25.9 ± 8.9 nM	27.4 ± 16.8 nM	NCDI	NCDI
Galα1-3Gal	NCDI	NCDI	NCDI	160.3 ± 30.0 nM
AsialoGM1	NCDI	NCDI	109.1 ± 45.6 nM	11.02 ± 3.9 μM
Maltose	NCDI	NCDI	NCDI	NCDI

a NCDI, no concentration-dependent interaction up to 100 μM maximum concentration.

### Glycan binding analysis of fimbriae associated with colonization of the upper urinary tract.

P fimbriae are strongly associated with E. coli strains that cause pyelonephritis (17) due to their capacity to bind to the α-d-galactopyranosyl-(1-4)–β-d-galactopyranoside receptor epitope in the globoseries of glycolipids found in human kidneys and on erythrocytes (15, 41). P fimbriae recognize their receptors via the tip-located PapG adhesin, which exists as three distinct alleles (PapGI, PapGII, and PapGIII) that bind with different affinity to Galα[1-4]Galβ epitopes; PapGI, PapGII, and PapGIII bind preferentially to membrane-associated GbO3, GbO4, and GbO5, respectively (15), while the three variants exhibit similar binding specificity when these glycosphingolipids are affixed to an artificial surface (41). Here, MS428 was used as a host strain and transformed with plasmids harboring P-fimbria genes containing either the *papGI* (pRHU845) or *papGII* (pPIL110-35) allele. Glycan array analysis of PapGI and PapGII demonstrated binding to terminal galactose, including α1-3Gal, α1-4Gal, and β1-3Gal structures (Fig. 1 and Data Set S1), although not as many as previously described (15, 17, 41). Based on the published studies (15, 17, 41), we could have expected up to 50 structures on the array to have showed binding rather than the fewer than 10 that were observed. Recently, it was demonstrated that glycan arrays are an imperfect tool for the analysis of glycan binding by proteins owing to the importance of glycan presentation (42). Grant et al. demonstrated that different chemical attachments to the array alter the recognition of the presented glycan (42). It is important to note that previous studies of PapG used ceramide-linked glycans, with many of the glycans on the array utilized here mimicking glycoprotein/extended glycan presentation rather than lipid-linked glycoconjugates. In fact, Stromberg et al. previously noted that the presentation of the same glycoconjugates in different membrane environments can alter the recognition of PapG fimbriae to saccharide structures (41). We also attempted to characterize the binding properties of the PapGIII adhesin by transforming MS428 with the *papGIII*-containing pJFK102 plasmid; however, the recombinant strain exhibited nonspecific binding to the ethanolamine-blocked glass surface of the array, preventing the precise elucidation of specific glycan interactions.

F1C fimbriae are expressed by 14 to 30% of extraintestinal E. coli (ExPEC) strains of urinary tract infection (UTI) origin (43, 44) and mediate binding via a tip-located FocH adhesin to galactosylceramide receptors on epithelial cells in the kidneys, ureters, and bladder as well as globotriaosylceramide receptors in the kidneys (21, 45). MS428 was transformed with plasmid pPKL143 (encoding the F1C fimbria cluster), and binding was examined using the glycan array. Binding to nine structures on the array was observed, six terminal α/βGal/GalNAc structures (glycan identifiers [IDs] 2C, 85, 262, 382, and 504; Data Set S1), two α2-6 sialylated structures [glycan ID 10O, Neu5Acα2-6Galβ1-4GlcNAcβ1-3Galβ1-4Glc, and glycan ID 627, Neu5Acα2-6Galβ1-4GlcNAcβ1-2Man)_2_-β1-3,6-Manβ1-4GNβ1-4GNβ-sp4], and two glycosylaminoglycan fragments, one from hyaluronan (glycan ID 13G) and the other from heparin (glycan ID 12L). Although the set of interacting glycans for fimbria F1C does not perfectly overlap the ganglioside and lactoceramide binding previously identified in the literature (21, 45), due likely to the same presentation issues observed for PapG fimbriae, binding to several previously identified structures, including the high-affinity structure asialoGM1, was observed. SPR analysis revealed MS428(pPKL143) cells bound strongly to asialoGM1, with a *K_D_* of 109 nM (Table 1).

### Glycan binding analysis of other E. coli CU fimbriae.

A further four CU fimbriae were examined to define their specific interacting glycan receptors. The meningitis-associated and temperature-regulated (Mat) fimbriae were first identified in the O18:K1:H7 clonal group (ST95) of NMEC (46). Subsequent work showed these fimbriae are also produced by other types of E. coli, including diarrheagenic strains, leading to their renaming as the E. coli common pilus, or ECP (47). Here, the Mat/ECP genes were PCR amplified from the UPEC reference strain CFT073 (ST73), cloned into plasmid pUC19, and transformed into MS428 to generate the strain MS428(pMAT). Whole MS428(pMat) cells expressing Mat/ECP fimbriae bound to four very different glycans on the array, Galα1-6Glc (glycan ID 83), Asialo-G_M1_ (Galβ1-3GalNAcβ1-4Galβ1-4Glc; glycan ID 1F), blood group B (Fucα1-2(Galα1-3)Gal; glycan ID 226), and GlcNAcβ1-6Galβ1-4GlcNAc (glycan ID 253). In the literature, the only other glycan targets identified for Mat/ECP fimbriae are plant (1→5)-α-linked l-arabinosyl residues and longer chains of arabinan (48). These structures are not on the glycan array used in this study and not present in animal hosts, suggesting further work is required to properly understand the receptor specificity of Mat/ECP fimbriae.

Yqi fimbriae (also referred to as ExPEC adhesin I; EA/I) are an uncharacterized CU fimbria type found predominantly in E. coli isolates from phylogroup B2 (49), which comprises extraintestinal E. coli associated with urinary tract, bloodstream, central nervous system, and avian origins. In avian-pathogenic E. coli (APEC), Yqi fimbriae are associated with adhesion and colonization of the lungs of chickens during infection (49, 50). Here, the *yqi* fimbrial gene cluster was amplified by PCR from UPEC strain CFT073, cloned into the expression vector pBAD/Myc-HisA, and transformed into MS428 to generate the strain MS428(pYqi). In the glycan array analysis, whole MS428(pYqi) cells expressing Yqi fimbriae bound to an asymmetrical biantennary glycan structure (Data Set S1; glycan ID 490) with a terminal β-galactose on one arm and a β-*N*-acetylglucosamine on the second, a terminal β-GlcNAc (Data Set S1; glycan ID 250), indicating some preference for terminal β-GlcNAc structures. Binding was also observed to three fucosylated structures; including Lewis A (Data Set S1; glycan ID 233), a blood group B glycan (Data Set S1; glycan ID 360), and α-Gal-Lewis X (Data Set S1; glycan ID 364). All of the structures recognized by Yqi are broadly expressed across tissue types and host species (51–53), with blood groups and Lewis antigens a common target of pathogens (54).

The receptor binding profile of two CU fimbriae from enterotoxigenic E. coli (ETEC) that mediate adhesion to porcine intestinal epithelial cells and contribute to ETEC diarrhea in neonatal and postweaning in piglets was examined using our whole-cell recombinant expression–glycan array system, namely, F4ac (K88ac) and F5 (K99) fimbriae (55, 56). Whole MS428(pK88-AC) cells expressing F4ac fimbriae bound to a range of α- and β-linked terminal galactose glycans with a preference for glycans containing a Galβ1-3GlcNAc core (Fig. 1 and Data Set S1). The binding to terminal Galα1-3 glycans by F4ac fimbriae indicates the recognition of nonhuman glycans, consistent with their disease association (26, 27). These Galα1-3Galβ1-3GlcNAc glycans are commonly expressed in the cells of all mammals except humans and old world monkeys (57). SPR analysis determined a binding affinity of 160 nM to α1-3-galactose and 11.2 μM to asialoGM1, supporting the observation that F4ac fimbriae interact with high affinity to terminal α1-3-galactose structures. Unfortunately, a direct comparison between the binding of F4ac and another F4 variant fimbriae, F4ab, could not be made, as MS428(pK88-AB) cells bound nonspecifically to the ethanolamine-blocked glass surface of the array, thereby preventing the precise elucidation of its glycan affinity profile. Whole MS428(pK99) cells expressing F5 fimbriae only bound to one glycan on the array, Neu5Acα2-3Galβ1-3GlcNAc (Fig. 1 and Data Set S1). This is a common glycan found in the gastrointestinal tract and lungs of pigs (58, 59) and is similar to the previously defined F5-interacting glycan Neu5Gcα2-3Galβ1-4Glc-ceramide (27, 60), which was not present on the glycan array. No binding was detected to the terminal disaccharide Neu5Gcα2-3Galβ (glycan number 206) (Fig. 1 and Data Set S1). Taken together, the data presented here, together with previous data in the literature, suggest these fimbriae bind with a preference to sialic acids containing α2-3 linkages but can also bind to N-acetyl- and N-glycyl-neuraminic acids, both of which are present in pigs (58, 59).

### Conclusions.

The capacity to rapidly screen and measure adhesin-glycan interactions provides new opportunities to understand bacterium-host tissue tropism and creates a platform for the development of new therapeutics to prevent bacterial colonization and disease. Indeed, given the current scenario of rapidly increasing antibiotic resistance, together with the dearth of new antibiotics in the developmental pipeline, such alternative treatment approaches are urgently required. The best examples of anti-adhesive therapeutics are mannosides, high-affinity mannose analogues that bind to the FimH adhesin of type 1 fimbriae, and reduce E. coli colonization of the bladder and colon (30, 33, 61, 62). Atomic detail describing the binding of mannoside derivatives to FimH has led to their optimization for enhanced activity, potency, and oral bioavailability and demonstrated their therapeutic efficacy in animal models of experimental UTI (34, 63). Other small-molecule receptor analogues have also been developed, with high-affinity galactosides that block E. coli Fml fimbria-mediated binding to GalNAc-containing receptors in the urinary tract also showing promise for the treatment of bladder and kidney infection in experimental mice (64, 65). The work described here presents a flexible, rapid, and scalable system to define novel adhesin-glycan interactions that underpin bacterial colonization and could be used to identify lead glycan structures for the development of new therapeutics.

## MATERIALS AND METHODS

### Strains, plasmids, and culture conditions.

E. coli strains and plasmids used in this study are listed in Table 2. The heterologous expression of specific fimbriae was achieved by transformation of the *fim*-negative MS428 strain with plasmids containing genes encoding type 1, P, F1C, Mat/ECP, Yqi, F4ac (K88ac), or F5 (K99) fimbriae. E. coli strains were routinely cultured at 37°C on solid or in liquid lysogeny broth (LB) medium (66) or liquid M9 minimal medium (42 mM Na_2_HPO_4_, 22 mM KH_2_PO_4_, 9 mM NaCl, 18 mM NH_4_Cl, 1 mM MgSO_4_, 0.1 mM CaCl_2_, and 0.2% [wt/vol] glucose). Where appropriate, media were supplemented with ampicillin (100 mg ml^−1^), kanamycin (100 mg ml^−1^), or chloramphenicol (25 mg ml^−1^). FimH^LD^ was expressed and purified as previously described (35).

**TABLE 2:** E. coli strains and plasmids used in this study

Strain/plasmid	Description	Reference or source
Strain		
MS428	MG1655 Δ*fim*	36
Plasmids		
pPKL4	Type 1 fimbria gene cluster from E. coli PC31 in pBR322	71
pPKL143	F1C fimbria gene cluster from E. coli AD110 in pBR322	72
pRHU845	P fimbria gene cluster (PapGI) from E. coli J96 in pACYC184	73
pPIL110-35	P fimbria gene cluster (PapGII) from E. coli AD110 in pACYC184	74
pJFK102	P fimbria gene cluster (PapGIII) from E. coli J96 in pBR322	73
pK88-AC	F4ac (K88ac) gene cluster in pBR322	Gift from Per Klemm
pK99	F5 (K99) gene cluster in pBR322	Gift from Per Klemm
pMAT	Mat gene cluster from CFT073 in pBR322	This study
pYqi	Yqi gene cluster from CFT073 in pBR322	This study

### Glycan array analysis of purified FimH protein.

Glycan array slides were printed using SuperEpoxy 3-activated substrates as previously described in Waespy et al. (67). The glycan arrays were preblocked with 1% bovine serum albumin in phosphate-buffered saline (PBS) for 15 min. The glycan array binding experiments were performed using 1 μg of FimH^LD^ protein in 65 μl and analyzed as previously described in Shewell et al. (68).

### Glycan array analysis of recombinant E. coli strains expressing different fimbriae.

E. coli MS428 strains harboring plasmids encoding different CU fimbriae were grown in M9 minimal media at 37°C to an optical density at 600 nm (OD_600_) of 0.5 to 0.6, at which time Bodipy methyl ester TR fluorescent label was added at a final concentration of 20 μM. Labeling was carried out by incubating the cells at 37°C in the dark with gentle shaking for 60 min.

Glycan arrays were produced as previously described (67, 69) and applied to the arrays as described in Wurpel et al. (70). A total of 500 μl of the labeled bacterial mix was added to a 65-μl gene frame as a bubble and left to incubate in the dark for 20 min. Slides were washed three times with PBS and fixed with 4% formaldehyde prior to being spun dry. Slides were scanned and analyzed as previously described (68) and as outlined in the MIRAGE compliance table (see Table S1 in the supplemental material).

10.1128/mBio.03664-20.2 TABLE S1 MIRAGE compliance information for the screening of E. coli fimbriae on the Institute for Glycomics glycan array. The supplementary glycan microarray document is based on MIRAGE guidelines (10.1093/glycob/cww118). Download Table S1, DOCX file, 0.02 MB. Copyright © 2021 Day et al. 2021 Day et al. https://creativecommons.org/licenses/by/4.0/ This content is distributed under the terms of the Creative Commons Attribution 4.0 International license.

### SPR analysis of bacterial fimbrial proteins.

The interactions between the FimH^LD^ protein and mannose glycans were analyzed using surface plasmon resonance (SPR) as described by Shewell et al. (68), with the following modifications. Proteins were immobilized onto a CM5 chip at pH 4.0 and flow rate of 5 μl/min for 600 s, with an ethanolamine blank flow cell as a control. Glycans were tested between 1.6 nM and 1 μM. All data were double reference subtracted. SPR of whole bacteria was performed using a Biacore T200 system and C1 series S sensor chips (GE Healthcare Life Sciences) as previously described (40). All flow cells (1 to 4) were prepared for immobilization per the manufacturer's instructions, and bacteria were flowed at an OD_600_ of 0.2 in a pH 5.0 acetate buffer and immobilized at a flow rate of 5 ml/min for 720 s. MS428 was immobilized onto flow cell 1 as the negative control. Glycan was flowed over between 1.6 nM and 100 μM glycans using single-cycle kinetics. The dissociation constant (*K_D_*) of the interactions was obtained using the Biacore T200 evaluation software package (GE Healthcare Life Sciences).
