# Supplementary material for: Discovery of Bacterial Fimbria–Glycan Interactions Using Whole-Cell Recombinant Escherichia coli Expression
Source: mBio. 2021 Feb 23;12(1):e03664-20. doi: 10.1128/mBio.03664-20 (PMC8545135; doi:10.1128/mBio.03664-20)
Supplement: TABLE S1 [file mbio.03664-20-st001.docx]

**Supplementary Table S1 -** Supplementary glycan microarray document based on MIRAGE guidelines DOI: 10.1093/glycob/cww118.

| **Classification** | **Guidelines** |
| --- | --- |
| 1. **Sample: Glycan Binding Sample** | |
| Description of Sample | Sample names:  *Escherichia coli* MS428 (fimbrial locus knockout strain) with/without plasmid expressing different fimbrial clusters as outlined in Table 2.  Origin: *Escherichia coli*.  FimH^LD^ – ligand binding domain of the Type 1 fimbriae.  Origin: *Escherichia coli;* produced as a recombinant protein in *E. coli*.  Method of preparation:  The preparation of samples is explained in the Materials and Methods section. |
| Sample modifications | FimH^LD^ is a histidine-tagged protein. |
| Assay protocol | Please see *Materials and Methods*. |
| **2.** **Glycan Library** | |
| Glycan description for defined glycans | Glycans in this study are listed in Supplementary Dataset 1 and is a published library in doi: 10.1371/journal.pntd.0004120. |
| Glycan description for undefined glycans | N/A. |
| Glycan modifications | Glycans were prepared in one of two ways for printing:  1. Glycans (with IDs in number/letter format; e.g. 1A, 4C, 7K) were sourced commercially from Dextra Laboratories, Elicityl and Carbosynth and were made into glycoamines using the protocol published in Day et al 2009 (doi: 10.1371/journal.pone.0004927).  2. Glycans (with IDs in number only format) were obtained from Prof. Nicolai Bovin and were modified with spacers as per DOI: 10.1073/pnas.0407902101. The library of these glycans was first published in DOI: 10.1016/j.molimm.2009.06.010. |
| 1. **3.** **Printing Surface; e.g., Microarray Slide** | |
| Description of surface | Epoxy activated glass microarray slides. |
| Manufacturer | ArrayIt SuperEpoxy 3 (SME3). |
| Custom preparation of surface | N/A. |
| Non-covalent Immobilisation | N/A. |
| **4. Arrayer (Printer)** | |
| Description of Arrayer | SpotBot® Extreme Protein Microarray Spotter (ArrayIt, California, USA). |
| Dispensing mechanism | Contact printing using 946NS6 pins with a 6 pin in a 3 columns x 2 rows configuration. |
| Glycan deposition | Approximately 1.8 nl per spot is printed according to manufactures guidelines.  Glycans were at 500 µM in 50:50 DMF:DMSO. |
| Printing conditions | Arrays were printed with dehumidification at a maximum humidity of 60% relative humidity (Standard laboratory starting humidity of 75-90%) at 22ºC. Glycans were left to react with the slide for at least 8 hours after the print was completed. |
| 1. **5.** **Glycan Microarray with “Map”** | |
| Array layout | The array consists of a single array of glycans split between 6 pins (3 columns x 2 rows) with 4500 μm row and column spacing. Each pin printed a 20 columns x 16 rows with 200 μm spot spacing (centre to centre) with a minimum spot size of 100 μm. Each sample is printed in quadruplicate with each of the 6 print areas including at least three negative control samples (print solution only) and two positive control samples consisting of one sample of fluoroscienamine and one sample of a mixture of rabbit anti-mouse antibody labeled with Alexa 555 and Alexa 647. Positive controls provide proof of successful immobilization of the amine reagents and provides for orientation for analysis. The antibodies can also provide controls for secondary antibodies used in experiments (if applicable). |
| Glycan identification and quality control | Arrays are quality controlled by a range of measures. 1. Each printed array is post print scanned to confirm deposition of the glycans on the array surface prior to neutralization of the remaining slide surface. 2. Post neutralized slides are scanned again to monitor for remaining autofluorescence. 3. Slides are assayed with fluorescently labeled lectins: WGA-Texas Red (EY Laboratories) and ConA-FITC (EY Laboratories). |
| 1. **6. Detector and Data Processing** | |
| Scanning hardware | ProScanArray 4 laser (488 nM, 532 nM, 595 nM, 647 nM) scanner (Perkin Elmer). |
| Scanner settings | Scanning resolution: 10 µM.  Laser channels: 595 nm excitation / 625nm emission filter for the bacterial analysis and 555nm /568 nm for the protein analysis.  PMT: 70% gain.  Scan powers: 100% laser power. |
| Image analysis software | ScanArray Express (Perkin Elmer). |
| Data processing | Data was exported as a CSV file and exported to Microsoft Excel. |
| **7.** **Glycan Microarray Data Presentation** | |
| Data presentation | Data is presented as yes/no binding in Figure 1. Data including glycan identification and analysed values of above background binding (see 8. below) are presented in Supplementary dataset 1. |
| 1. **8.** **Interpretation and** **Conclusion from Microarray Data** | |
| Data interpretation | We only use glycan arrays as a yes/no binding tool. Due to this we look only at binding that is unambiguously above background vs lack of binding above background. Average background + 3x standard deviation of the background of 20 sets of 4 spots of DMF:DMSO only spots is applied to determine if binding observed is significantly above background. Only spots with values equal to or greater than this value were considered as binding from data of any tested slide. These values are slide dependent. |
| Conclusions | Fimbrial proteins can be successfully analyzed without the need to purify the adhesin using expressing of the fimbrial cluster in MS428. There are limitations for the analysis of some of the fimbrial adhesins tested with the array surface and spacer presentation resulting in lack of binding to some of the expected glycans. |
